# Fluorescence engineering in metamaterial-assisted super-resolution localization microscope

**DOI:** 10.1515/nanoph-2022-0751

**Published:** 2023-03-31

**Authors:** Kyu Ri Choi, Shilong Li, Igor Ozerov, Frédéric Bedu, Dong Hee Park, Bin Chan Joo, Jeong Weon Wu, Síle Nic Chormaic, Yeon Ui Lee

**Affiliations:** Department of Physics, Chungbuk National University, Cheongju, Chungbuk 28644, South Korea; Light-Matter Interactions for Quantum Technologies Unit, Okinawa Institute of Science and Technology Graduate University, Onna, Okinawa 904-0495, Japan; Aix-Marseille University, CNRS, CINaM UMR 7325, AMUTech, Marseille, France; Department of Physics, Ewha Womans University, Seoul 03760, South Korea

**Keywords:** enhanced fluorescence, metamaterials, Purcell effect, super-resolution imaging

## Abstract

Single-molecule localization microscopies have gained much attention for their efficient realization of a sub-diffraction-limit imaging with the resolution down to the 10-nm range. In contrast to conventional localization microscopes, which rely on particular fluorescent probes in specific conditions, metamaterial-assisted super-resolution microscopies can be implemented with any fluorescent dye under general conditions. Here, we present a systematic study of fluorescence engineering in metamaterial assisted localization microscopy by using cyclic group metasurfaces coated with a fluorescent film. Tailored variations are clearly demonstrated in both the photoluminescence intensity and the photobleaching lifetime of fluorophores based on the spatially varied Purcell effect near the metasurfaces. The enhanced emissions and blinking dynamics of the fluorophores on these metasurfaces lead to an increased signal-to-noise ratio, and therefore give rise to a super-resolution localization image with 0.9-nm localization accuracy. Our results are not only beneficial for super-resolution localization imaging but also push the control of light–matter interactions beyond the diffraction limit.

## 1 Introduction

Single-molecule localization microscopy (SMLM) is a powerful super-resolution imaging technique with a spatial resolution of typically ∼20–50 nm [1]. Distinguished from other super-resolution microscopies such as stimulated emission depletion microscopy [2] and structured illumination microscopy [3], the spatial resolution of SMLM is not limited by the wavelength of light but by the signal-to-noise ratio (SNR)—this is because the SMLM super-resolution image is the superposition of temporally non-overlapped fluorescence images of all individual fluorophores. To match the desired temporal separation of fluorescence, specific fluorophores with at least two emission states, such as photoswitchable dyes, photoactivatable fluorescent proteins, photoconvertible fluorescent proteins, and certain spontaneously blinking dyes, can be used for SMLM in a particular switching-inducing environment such as pH, buffer, or UV irradiation [1, 4]. Amongst these SMLM fluorophores, spontaneously blinking fluorophores have the lowest or even no restrictions on the emission-state switching and can work at a low excitation power density to mitigate photo-induced damages. However, their weak spontaneous emission, low spontaneously blinking rate, and unavoidable photobleaching limit the SNR and the final SMLM imaging resolution.

In a different context, the fluorescence of spontaneously blinking dyes can be engineered by modifying the local electromagnetic environment with plasmonic nanostructures or metamaterials: (*i*) The photoluminescence (PL) intensity of dye molecules has been enhanced near a plasmonic nanoantenna due to the increased excitation rate, the enhanced spontaneous decay rate (Purcell effect), and the improved emission directionality of such a molecule-nanoantenna system [5–8]; (*ii*) The blinking of spontaneously blinking dyes has been accelerated [9] in a plasmonic nanocavity although the underlying physics is still under debate [10]—possible mechanisms include intensified fluctuations in an enhanced electric field [11, 12], or even the random formation and disappearance of quantum-confined emitters and crystal defects near metal surfaces [13]; (*iii*) The photostability of dye molecules has been improved on top of a low-loss organic metamaterial film due to suppressed photobleaching, which is also a result of enhanced spontaneous emission described by the Purcell effect [14].

With these fluorescence-engineered spontaneously blinking dyes, advanced SMLM imaging approaches are straightforward. For example, SMLM imaging with a fluorescence enhancement of 480% and an increased blinking rate has been demonstrated by using surface plasmon-coupled emission substrates [9, 15], which significantly reduced the required excitation power density. Another astonishing achievement is that, combined with the Brownian motion of single molecules in a fluidic chamber, SMLM imaging of the local field of plasmonic hotspots has been performed with an accuracy as low as 1.2 nm [16, 17]. Nevertheless, previous works on spontaneously blinking dye-based SMLM imaging of plasmonic nanostructures and metamaterials [9, 16], [17], [18], [19] were focused on single or periodic structures, generally in an immersion liquid, with less attention on the fluorescence photostability—one of the key aspects in fluorescence engineering.

In this Letter, we use fluorescent film coated aperiodic and asymmetric metasurfaces to show both fluorescence engineering and super-resolution imaging in an SMLM process without using any immersion liquid. Metamaterial-assisted localization microscopy (MALM) is thus realized. Our experimental results directly map out the nanoscale interactions between fluorophores and their local electromagnetic environment. We believe that our study will promote this versatile nanoscopic tool in a broad range of applications.

## 2 Results

**Figure 1:**
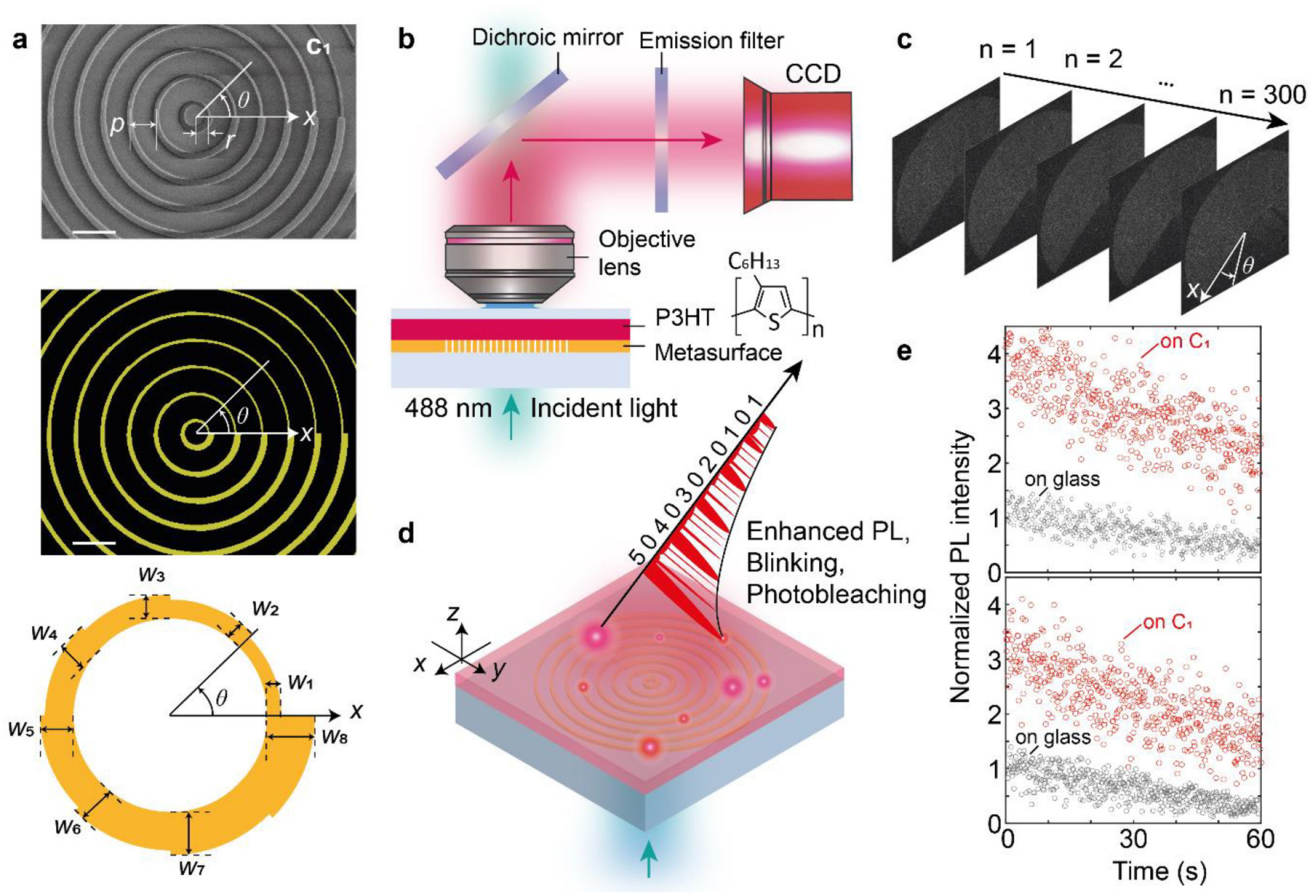
Metamaterial assisted localization microscopy (MALM). (a) Cyclic group C_1_ metasurface adopted for the MALM. Scanning electron microscope (SEM) image of the fabricated C_1_ metasurface (top panel) shows the first few spiral rings with the radius of the *N*th ring *r* = (*pN* – 300) nm, where the separation *p* = 600 nm, as designed (middle panel). Each spiral ring is composed of 8 arcs with widths from *W*_1_ = 45 nm to *W*_8_ = 150 nm with a 15-nm increment (bottom panel). Each arc functions as a nanoantenna. Scale bars: 1 μm. (b) Schematic illustration of the MALM setup showing its two main components: a fluorescence microscope and a metamaterial substrate coated with spontaneously blinking fluorophores. Here, the metamaterial substrate is the C_1_ metasurface (a), on top of which a P3HT film is spin-coated and provides the spontaneously blinked emissions. The P3HT film was excited by a 488-nm CW laser, and the emission was collected by an objective lens (40 × /0.75 NA) and then recorded by a CCD camera after passing through a 650-nm long-pass filter. (c) Diffraction-limited fluorescence blinking images used for the MALM super-resolution image reconstruction. There are 300 frames of images recorded at a rate of 5 frames per second (5 fps). (d) Fluorescence engineering in the MALM imaging process. Owing to the Purcell effect near the arc nanoantennas, the surrounding fluorescence processes are altered: Enhanced PL intensity, accelerated spontaneous blinking, and improved photostability. (e) Example of the engineered fluorescence at two pixel positions. Compared to the case when the P3HT film is coated on top of glass (gray dots), the altered fluorescence dynamics in the MALM is clearly visible (red dots).

In principle, the MALM demonstrated here works for any plasmonic metamaterials by using any fluorescent molecules or materials that spontaneously blink. To have a comprehensive view of interactions between the fluorophores and their local electromagnetic environment, we focused on an aperiodic and asymmetric metasurface, i.e. a metasurface with cyclic group symmetry [20]. Figure 1a shows the fabricated cyclic group C_1_ metasurface, which consists of a series of gold arc nanoantennas with an increased width along the angular direction and an increased length along the radial direction. As for the spontaneously blinking materials, P3HT [14, 21] was used and spin-coated on top of the C_1_ metasurface (Figure 1b, see Methods for details); PL spectra of the produced P3HT films are given in Figure S1a (Supporting Information S1). Fluorescence microscope (K1-Fluo, Nanoscope Systems) was adopted to implement the MALM, as schematically shown in Figure 1b. As an SMLM approach, to obtain the super-resolution image with the MALM, temporally non-overlapped, fluorescence images are required; therefore, 300 frames of diffraction-limited images were recorded (Figure 1c). Owing to the enhanced PL intensity [5–8], accelerated spontaneous blinking [10, 13], and improved photostability [14] of the P3HT fluorescence by the C_1_ metasurface in the MALM (Figure 1d), the MALM super-resolution image with a higher localization accuracy—compared to a conventional SMLM—was obtained, as demonstrated below. Here, in Figure 1e, we show an example of the engineered P3HT fluorescence at two pixel positions of the 300 diffraction-limited images. Compared to the case when P3HT is coated on top of glass, an increased emission intensity and its fluctuation in the MALM are clearly visible. Since these fluorescence dynamics are position dependent over the C_1_ metasurface, various MALM super-resolution images made of different dynamics parameters, such as the photobleaching lifetime and Purcell factor, become readily available.

**Figure 2:**
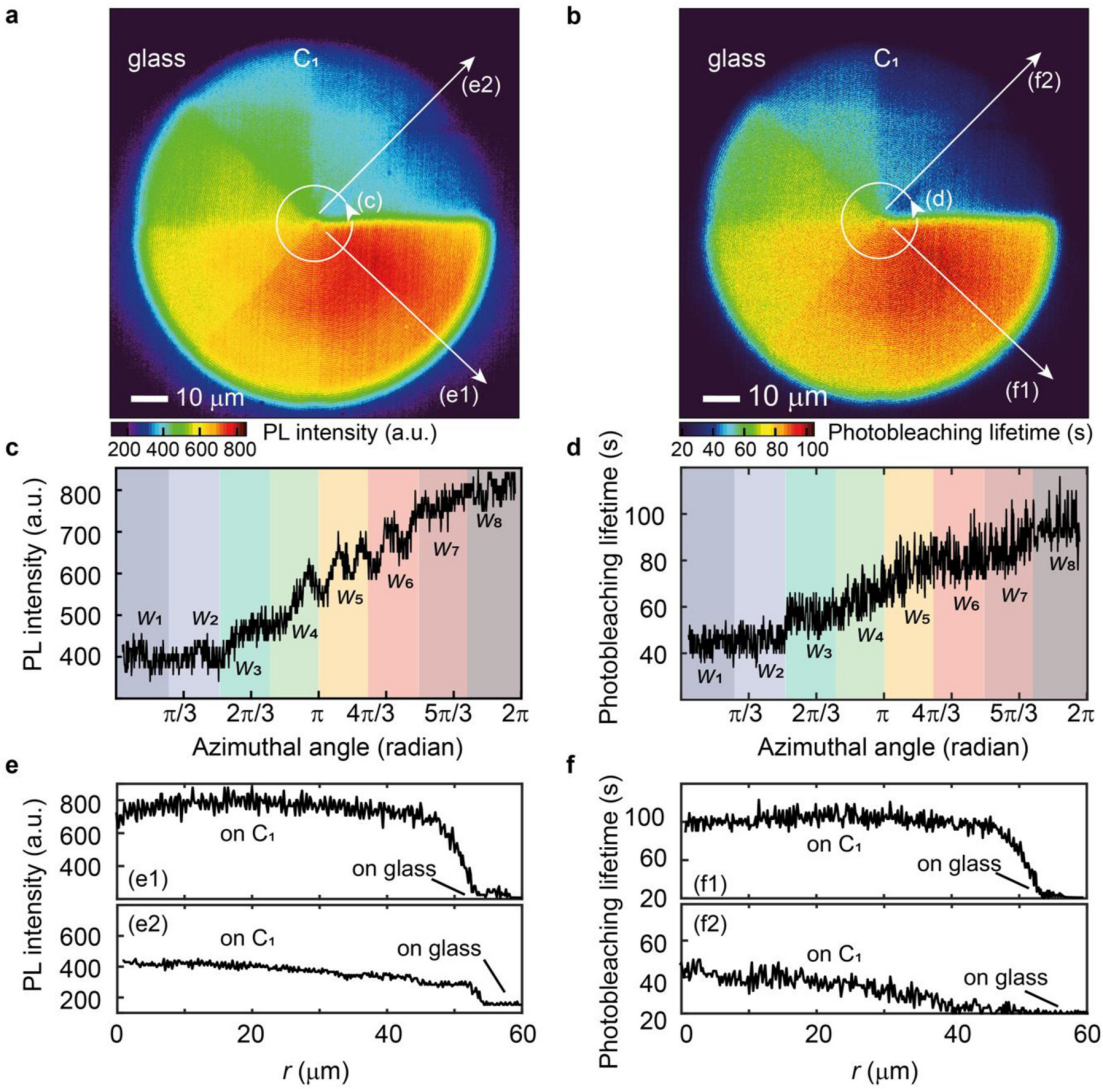
Fluorescence engineering with MALM metasurface substrate. (a, c, e) PL intensity image of the P3HT-coated C_1_ metasurface. Enhanced PL intensity is clearly visible (a). It directly shows that this intensity enhancement is determined by the width of the arc nanoantennas (c) and independent of their length (e). Note that the PL intensity of P3HT near the arc nanoantenna with a width of *W*_8_ = 150 nm (*W*_1_ = 45 nm) is about 4 (2) fold higher than that on the glass substrate. (b, d, f) Photobleaching lifetime image of the P3HT-coated C_1_ metasurface. Improved photostability is clearly visible (b). It directly shows that this stability improvement is determined by the width of the arc nanoantennas (d) and independent of their length (f). Note that the photobleaching lifetime of P3HT near the arc nanoantenna with a width of *W*_8_ = 150 nm (*W*_1_ = 45 nm) is about 5 (2) fold longer than that on the glass substrate.

With the aperiodic and asymmetric C_1_ metasurface, interactions between the P3HT fluorophore and its local electromagnetic environment in the MALM can be easily imaged. Figure 2a is the PL intensity image of the P3HT-coated C_1_ metasurface which directly shows that the PL intensity is enhanced, and this enhancement is tailored by the width (Figure 2c) of the gold nanoantennas. On the other hand, there is no significant spatial variation of the PL enhancement along the radial direction at a given polar angle (Figure 2e). Such a conclusion could also be obtained using a periodic metasurface, but different samples or different sample areas with varied widths and lengths are needed, which leads to the difficulty of making a fair comparison.

Photobleaching of dye molecules sets a fundamental limitation on fluorescence microscopes including SMLM [1, 14]. Recent efforts were made to attenuate the photobleaching of dye molecules with organic metamaterials through an enhanced spontaneous emission [14]. Therefore, the study of photostability in metamaterial-assisted SMLMs is crucial. To consider many different aspects of fluorescence engineering in metamaterial-assisted SMLMs, the fluorescence photostability in the MALM is investigated here by examining the photobleaching lifetime. As shown in Figure 1e, the photobleaching lifetime can be extracted by fitting the decaying PL intensity curve [22]. Figure 2b is the extracted photobleaching lifetime image of the P3HT-coated C_1_ metasurface. It shows directly that the photobleaching is suppressed, and the amount of suppression is determined by the width (Figure 2d) of the gold nanoantennas. In contrast, there is no significant spatial variation of the photobleaching suppression along the radial direction at a fixed polar angle (Figure 2f). The same conclusion is obtained for the enhanced PL intensity (Figure 2a,c,e), indicating that these two light–matter interaction processes have the same physics origin as the Purcell effect (Figure S1b–d in Supporting Information S1 as well as Figures S2 and S3 in Supporting Information S2). Such a clear demonstration highlights the advantage of this emerging nanoscopic tool to explore light–matter interactions.

**Figure 3:**
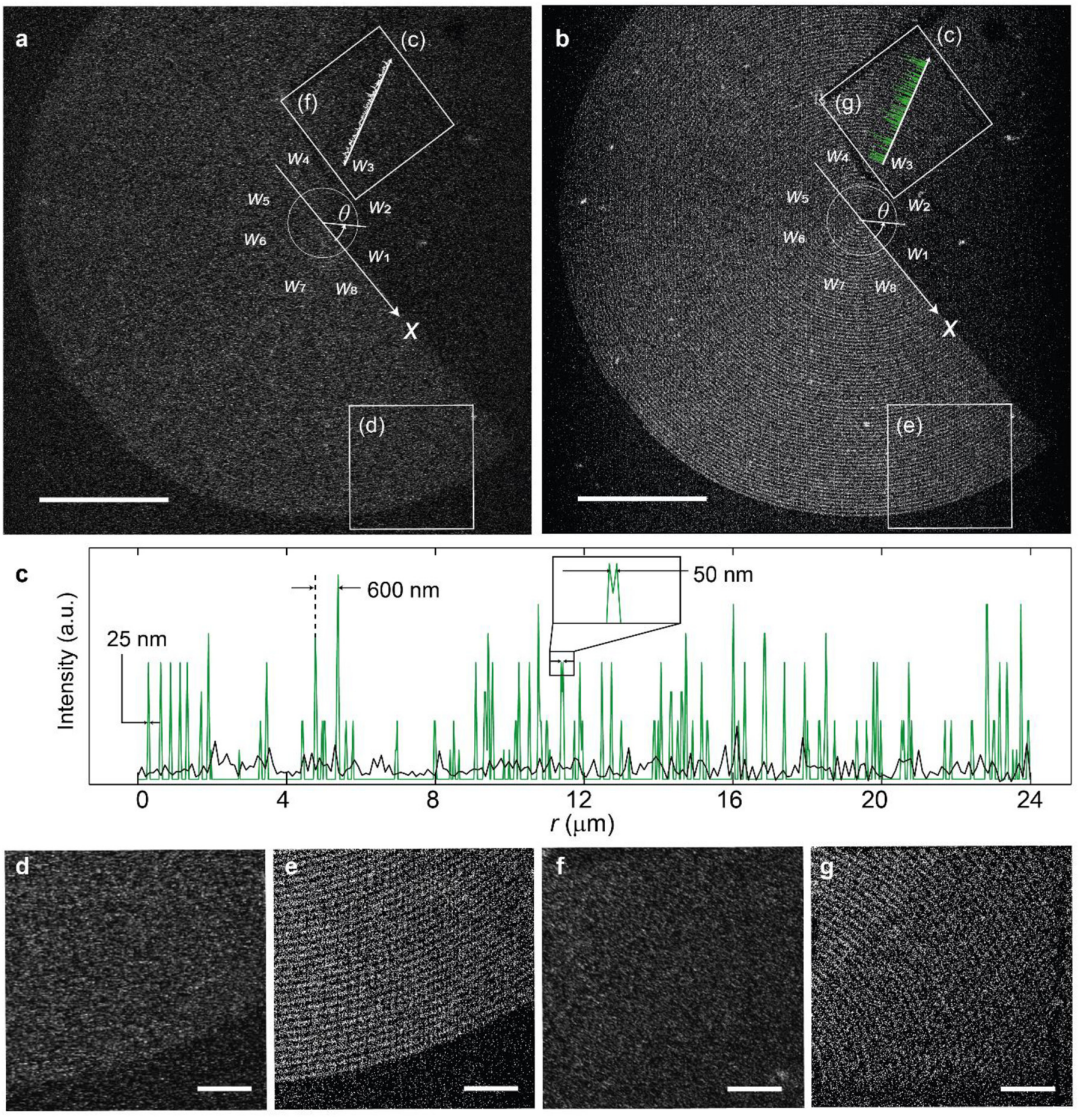
Super-resolution image of MALM. (a, b) Diffraction-limited image (a) and the MALM super-resolution image (b) of the P3HT-coated C_1_ metasurface. Scale bars: 25 μm. (c) Radial intensity distributions of the diffraction-limited image (black curve) and the MALM super-resolution image (green curve) along the lines indicated in (a) and (b), respectively. In addition to the sub-diffraction-limited resolution, the MALM super-resolution image also exhibits high contrast. (d, f) Magnified diffraction-limited images of two regions of interest. The spiral rings cannot be resolved. Scale bars: 5 μm. (e, g) Magnified MALM super-resolution images of the same regions of interest. The spiral rings are clearly visible. Scale bars: 5 μm.

The super-resolution imaging results using the MALM are summarized in Figure 3. Figure 3a,c,d,f show the last frame of the recorded 300 diffraction-limited images of the P3HT-coated C_1_ metasurface. Since the diffraction limit is ∼433 nm at the shortest detection wavelength of 650 nm, the gold nanoantennas (45–150 nm in width) cannot be resolved; the gap (450–550 nm) between them along the radial direction is also barely visible due to the low image contrast. Nevertheless, using all these 300 diffraction-limited images, the MALM super-resolution image was obtained via SMLM reconstruction algorithm [17, 23] (see Methods for details), and the results are given in Figure 3b,c,e,g. Now, the radial structure of the C_1_ metasurface is clearly visible with a high image contrast, and the image resolution is well beyond the diffraction limit (Figure 3c): Two localization peaks are apparent with a center-to-center distance of ∼50 nm, while a single localization peak has a full width at half maximum (FWHM) of ∼25 nm. Such a high-contrast super-resolution image makes the MALM useful in imaging applications where liquid solution is not allowed.

**Figure 4:**
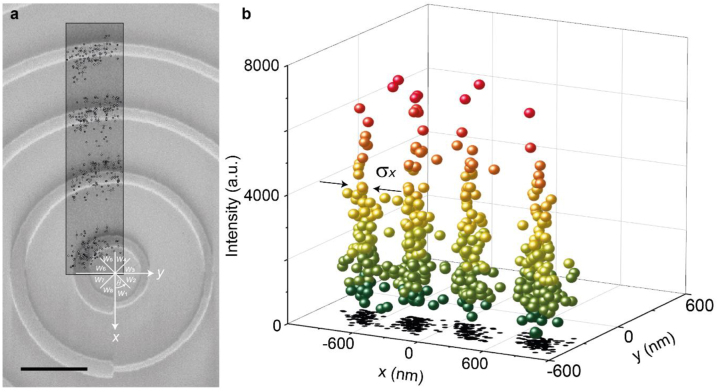
Localization performance of MALM. (a) Localization results overlapped with the corresponding SEM image. The error bars show the localization accuracy. Scale bar: 600 nm. (b) Localization results visualized as a scatter plot. The standard deviation *σ*_*x*_ shows the localization precision.

Figure 4 summarizes the accumulated localization events with subpixel-sized bins in a small sample region for clarity. There are four peaks resolved with a high contrast as shown before, and the standard deviation of these peaks is around 98 nm (Figure 4b) with a 0.9-nm localization accuracy (error bars in Figure 4a); it is noted that the achieved localization accuracy is comparable with previous findings in SMLMs that employed plasmonic nanoantennas [16, 17]. We overlapped the corresponding SEM image with the localization results as given in Figure 4a. It reveals that these single-molecule localization events due to the spontaneously blinked emissions of the P3HT film are prominent near the nanoantennas of the C_1_ metasurface. This super-resolution result confirms that the blinking of P3HT emission is accelerated by the C_1_ metasurface. Since both the enhanced PL intensity (Figure 2a,c,e) and improved photostability (Figure 2b,d,f) are due to the Purcell effect provided by the C_1_ metasurface, we believe that this super-resolution image is highly correlated with the spatial distributions of PL enhancement and photobleaching improvement.

**Figure 5:**
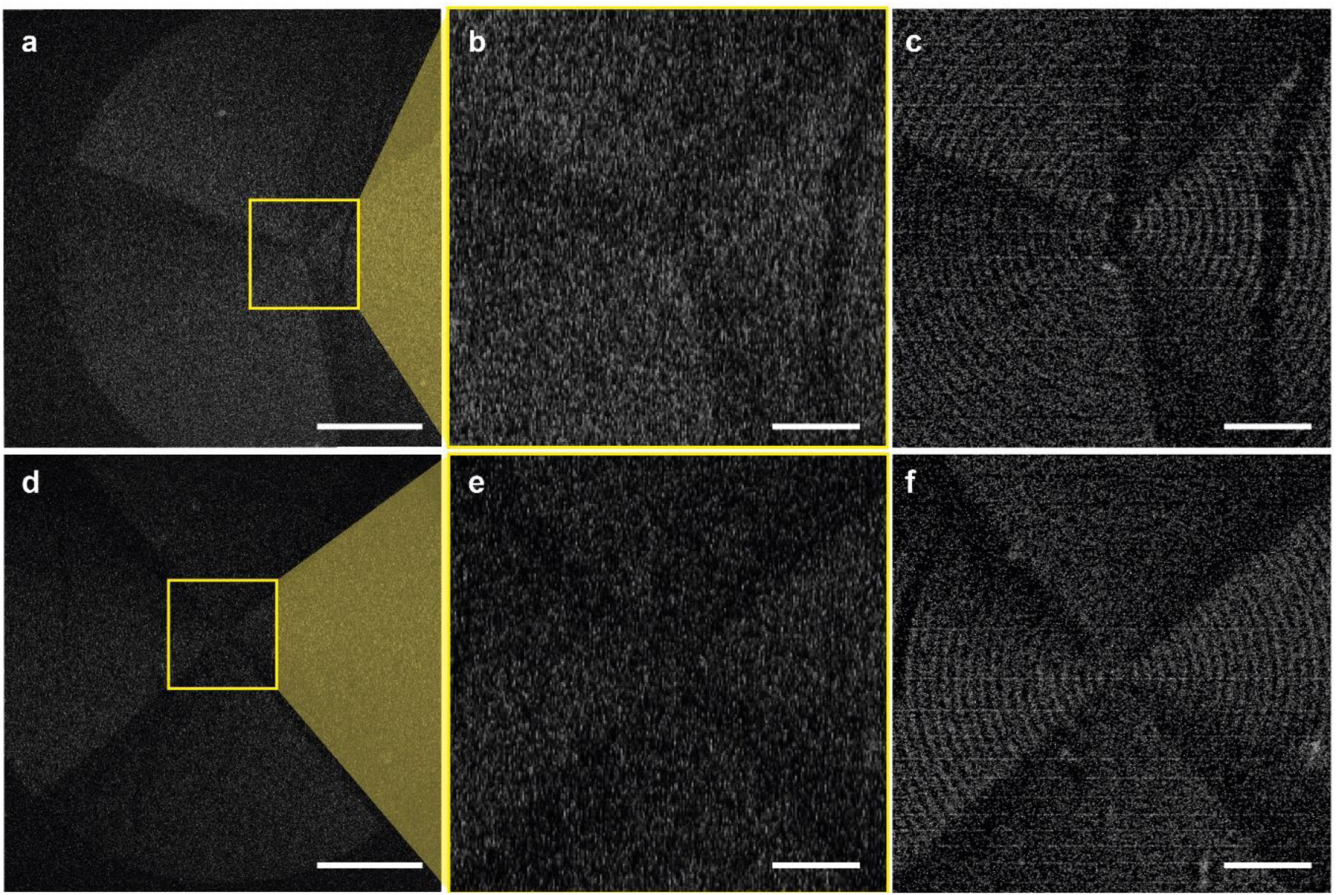
MALM super-resolution images of P3HT-coated C_3_ and C_4_ metasurfaces. (a–f) Diffraction-limited images (a and d), magnified diffraction-limited images of a region of interest (b and e), and the MALM super-resolution images (c and f) of the C_3_ and C_4_ metasurfaces, respectively. Scale bars: 25 μm (a, d) and 5 μm (b, c, e, f).

To examine the validity of the demonstrated MALM approach, P3HT-coated higher-order cyclic group metasurfaces [20], i.e. the odd-number C_3_ metasurface and the even-number C_4_ metasurface were imaged, and the results are summarized in Figure 5. The radial structure of both the C_3_ and C_4_ metasurfaces is visible only in their respective MALM super-resolution images, showing the effectiveness and usability of the MALM approach.

## 3 Conclusions

We have demonstrated a metamaterial-assisted localization microscope without using an immersion liquid, and presented a systematic study of fluorescence engineering in the localization process. Enhanced photoluminescence intensity, accelerated spontaneous blinking, and improved photostability due to the Purcell effect have been observed with various cyclic group metasurfaces. The engineered fluorescence leads to an increased signal-to-noise ratio, and enables super-resolution localization imaging with 0.9-nm localization accuracy. The demonstrated metamaterial nanoscopic approach can be extended to mapping out optical trapping force and hotspots near nanostructures at multiple wavelengths in a wide-field view and, therefore, would be very useful for imaging of trapped nanoparticles on plasmonic optical tweezers.

## 4 Methods

**Sample preparation.** Cyclic group metasurfaces were fabricated as described in Ref. [20]. 1-mm thick borosilicate glass substrates were cleaned with acetone and isopropyl alcohol and then dried with nitrogen flow, followed by oxygen radical plasma treatment in a barrel reactor to activate the glass surface. An e-beam resist layer and a conductive polymer layer were successively spin-coated, then e-beam lithography (Pioneer, Raith, Germany) was used to pattern the metasurfaces [20]. A 3-nm thick Cr layer (the seeding layer) and a 27-nm thick gold layer were deposited by thermal evaporation. The lift-off was done in ethyl lactate solution, followed by rinsing and nitrogen drying. 60-mg P3HT (Sigma Aldrich, average Mw ≈ 87,000 g·mol^−1^) was dissolved in 1-mL chlorobenzene, and then spin-coated on top of the fabricated metasurfaces for 60 s at 3000 rpm. The thickness of the P3HT layer is ∼540 nm measured with a dektak profiler.

**MALM super-resolution image reconstruction.** Open-source ThunderSTORM [23] plug-in module for ImageJ was used. The raw image sequence consists of 300 images with a resolution of 1024 × 1024 pixels. The fitting parameters used are listed as follows: SMLM image filtering was performed using an order 3 of a B-spline wavelet filter; localization was done by identifying the local maximum with std (Wave.F1) connectivity 8-neighborhood; a sub-pixel localization method was employed additionally with an integrated Gaussian in the PSF model; the fitting radius was 3 pixels; a normalized Gaussian overlay was used for visualization of the results. To avoid an overestimated plot resulting from the background noise, the intensity cutoff of a blinking event was defined two times above the standard deviation of background fluctuations in the reconstruction process.

## Supplementary Material

Supplementary Material Details
