# Supplementary material for: Fluorescence engineering in metamaterial-assisted super-resolution localization microscope
Source: Nanophotonics. 2023 Mar 31;12(13):2491–8. doi: 10.1515/nanoph-2022-0751 (PMC11501635; doi:10.1515/nanoph-2022-0751)
Supplement: Supplementary file 1 — Supplementary Material Details [file j_nanoph-2022-0751_suppl_001.docx]

Supporting Information

Fluorescence engineering in metamaterial-assisted super-resolution localization microscope

Kyu Ri Choi^1,‡^, Shilong Li^2,‡^, Igor Ozerov^3^, Frédéric Bedu^3^, Bin Chan Joo^1^, Dong Hee Park^1^, Jeong Weon Wu^4^, Síle Nic Chormaic^2^, Yeon Ui Lee^1,*^

^1^Department of Physics, Chungbuk National University, Cheongju, Chungbuk 28644, South Korea

^2^Light-Matter Interactions for Quantum Technologies Unit, Okinawa Institute of Science and Technology Graduate University, Onna, Okinawa 904-0495, Japan

^3^Aix-Marseille University, CNRS, CINaM UMR 7325, AMUTech, Marseille, France

^4^Department of Physics, Ewha Womans University, Seoul 03760, South Korea

^‡^These authors contributed equally to this work.

^*^Corresponding author email address: yeonuilee@cbnu.ac.kr

Contents

S1. Enhanced photoluminescence (PL) of P3HT film on C_1_ metasurfaces

S2. FDTD simulations of the PL enhancement

S1. Enhanced photoluminescence (PL) of P3HT film on C_1_ metasurfaces

Figure S1a shows the measured emission spectra of P3HT films on top of glass and C_1_ metasurface, respectively. As can be seen, the PL intensity is enhanced by the C_1_ metasurface in the wavelength range of 620–800 nm. Simulated PL intensity enhancement near the C_1_ metasurface was performed using the three-dimensional finite-difference time-domain (FDTD)^17,22,25^ method. The simulation models used are summarized in Fig. S1b and the results of Purcell factor are given in Figs. S1c and S1d. It shows that the Purcell effect is enhanced as the width of the arc nanoantennas increases up to 150 nm in the 620–800 nm wavelength range which contributes to the enhanced PL intensity and the increased photobleaching lifetime, as we observed in Fig. 2.

[fx6]

**Fig. S1 | Enhanced photoluminescence (PL) of P3HT film on C_1_ metasurfaces.** (**a**) PL spectra of P3HT films on top of glass and C_1_ metasurface, respectively. (**b**) Simulation models used. The 8 gold arcs of the C_1_ metasurface were modeled as 8 cylindrical nanoantennas. A dipole source was used in the simulations, and the results were averaged with two horizontal polarizations and one vertical polarization. (**c**,**d**) Simulation results of Purcell factor when a dipole is positioned on top of the plasmonic nanoantenna (c) and when it is located on the side (d).

S2. FDTD simulations of the PL enhancement

Figure S2 shows the phase diagrams of Purcell factor as a function of the wavelength and the nanoantenna width in different excitation cases. With these phase diagrams, the dispersion of various plasmonic modes is clearly discernible, allowing for direct identification of their excitation within the wavelength range of interest. In the 600–800 nm wavelength range, the first-order LSPR is dominant when the nanoantenna width is less than 150 nm, as mentioned above.

[fx7]

**Fig. S2 | Phase diagram of Purcell factor as function of the wavelength and the nanoantenna width.** (**a**–**d**) Simulation results of the Purcell factor when a dipole with (a) horizontal or (b) vertical polarization is positioned on top of the plasmonic nanoantenna and when the dipole with (c) horizontal or (d) vertical polarization is located on the side of the plasmonic nanoantenna. The dispersion of surface plasmon resonance (SPR) modes and localized surface plasmon resonance (LSPR) modes are highlighted by white dashed lines.

For completeness, more simulation results to evaluate the PL enhancement are summarized in Fig. S3. The PL enhancement can be defined as $\text{Γ}_{\text{exc}}P_{\text{r}}/P_{\text{r}0},$ where $\text{Γ}_{\text{exc}}=\left|E_{\text{exc}}\right|^{2}/\left|E_{\text{exc}0}\right|^{2}$ is the enhancement of the excitation field near the plasmonic nanoantenna at the excitation wavelength $\lambda_{\text{exc}}$ = 488 nm, $P_{\text{r}}/P_{\text{r}0}$ is the radiation enhancement due to the presence of the plasmonic nanoantenna in the detection wavelength $\lambda_{\text{det}}$ = 620–800 nm and the subscript ‘_0_’ represents the corresponding quantities in free space. To calculate the excitation rate enhancement (Fig. S3b), plane waves were incident from the glass substrate side. The electric field distributions were calculated at the excitation wavelength of 488 nm and the enhanced field intensity was obtained near the plasmonic nanoantenna. For the calculation of the radiative decay rate enhancement (Fig. S3c) and the nonradiative decay rate enhancement (Fig. S3d), the powers collected in the near and far fields were calculated using the corresponding transmission box monitors at the detection wavelength of 700 nm. In the end, FDTD calculation results of the PL enhancement are obtained and shown in Fig. S3e.

[fx8]

**Fig. S3 | FDTD calculations of the PL enhancement.** (**a**) Super-resolution MALM image. (**b**) Excitation rate enhancement at 488 nm. (**c**) Radiative decay rate enhancement at 700 nm. (**d**) Nonradiative decay rate enhancement at 700 nm. (**e**) PL enhancement.
